# A Case of Umbilical Invasive Extramammary Paget Disease With a High Tumor Mutational Burden Successfully Treated With Pembrolizumab

**DOI:** 10.7759/cureus.86186

**Published:** 2025-06-17

**Authors:** Akihiro Ishiguro, Kenichiro Mae, Ryokichi Irisawa, Hideaki Hirai, Kazutoshi Harada

**Affiliations:** 1 Dermatology, Tokyo Medical University, Tokyo, JPN; 2 Anatomic Pathology, Tokyo Medical University, Tokyo, JPN

**Keywords:** acute kidney injury, complete response, extramammary paget’s disease, immune-checkpoint inhibitors, immune-related adverse event, pembrolizumab, tumor mutation burden

## Abstract

Advanced extramammary Paget's disease (EMPD) is a rare skin cancer with no standard treatment. Anti-programmed cell death protein 1 (PD-1) agents are indicated for the treatment of EMPD with high tumor mutation burden (TMB) in Japan, but neither drug is necessarily effective. This report describes an 83-year-old male patient who presented with a macerated, reddish, 3-cm tumor on the umbilicus. Three years later, he developed metastasis of lymph nodes in the left inguinal region. He was diagnosed with EMPD. Positron emission tomography-computed tomography (PET-CT) revealed metastases to the lung and the bone. S-1 plus docetaxel therapy was begun, but the metastatic lesions were increased. A gene panel testing indicated a high TMB, so pembrolizumab was begun. After the second administration, Grade 3 acute kidney injury developed due to immune checkpoint inhibitors (ICIs). The treatment was discontinued after two administrations due to immune-related adverse events. All the metastases disappeared in seven months after the start of pembrolizumab administration. As far as we know, the present patient is the first case of advanced EMPD with a complete response to ICI. The efficacy of ICIs against EMPD awaits future investigation enrolling a larger patient cohort.

## Introduction

Extramammary Paget’s disease (EMPD) is a rare skin cancer that develops commonly in apocrine gland-rich skin; the genital, perianal, and axillary. Lesions in the genital most commonly affect Caucasian females, but lesions in the umbilicus have been reported predominantly in elderly Asian males [1,2]. EMPD tends to be indolent and is often identified as lesions in situ at the time of diagnosis. For advanced EMPD, chemotherapy with taxane-based treatments is used, but there is no established standard treatment [3]. Anti-programmed cell death protein 1 (PD-1) agents are indicated for the treatment of advanced skin cancer with a high tumor mutational burden (TMB) in Japan, but neither drug is necessarily effective for EMPD. As far as we know, the present patient is the first case of advanced EMPD with complete response (CR) to immune checkpoint inhibitor (ICI) monotherapy.

## Case presentation

**Figure 1:**
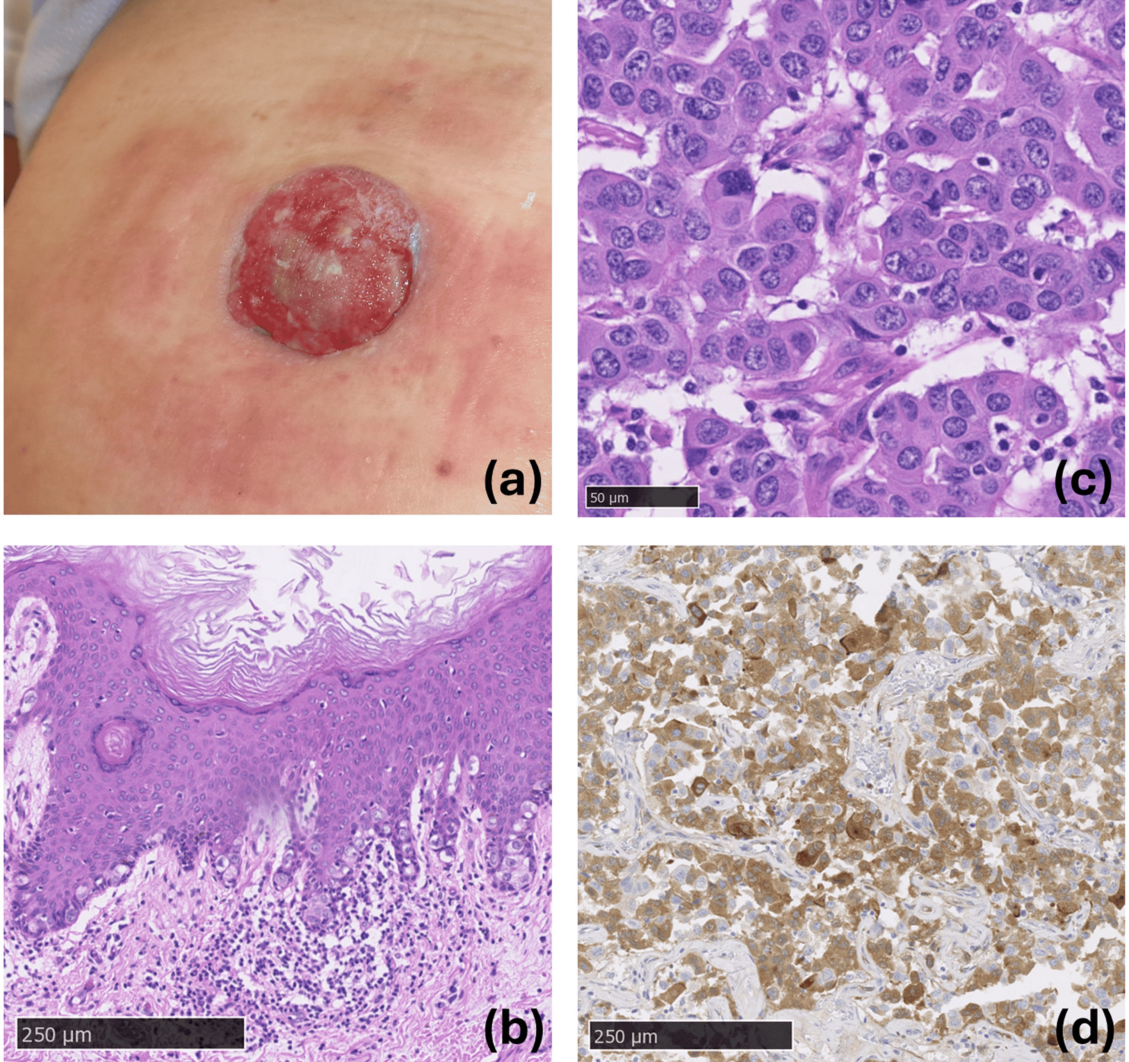
Clinical and histopathological features of the present patient. (a) Abdominal findings three years before the initial presentation. A cutaneous tumor with a diameter of approximately 3 cm was observed on the umbilicus. Erythema and infiltration were due to contact dermatitis in the surrounding areas. (b) Histopathological features around the umbilical nodule. Pagetoid spreading of the tumor cells with intracytoplasmic mucin was observed in the overlying epidermis (hematoxylin and eosin (HE) staining; scale bar: 250 μm). (c) Histopathological features of the umbilical nodule. High-power view revealed moderate to severe nuclear atypia and several mitotic figures of the tumor cells (HE staining; scale bar: 50 μm). (d) Immunohistopathological findings of the umbilical nodule. Diffuse and strong immunoexpression of GCDFP15 was seen (GCDFP15 immunostaining; scale bar: 250 μm).

An 83-year-old male Japanese patient presented with a reddish tumor on his umbilicus. His medical history included hepatocellular carcinoma (HCC), type 2 diabetes, and monoclonal gammopathy of indeterminate significance. Three years before the initial presentation, a physical examination revealed a macerated, reddish, 3-cm tumor on the umbilicus (Figure 1a). The tumor was totally excised with a 1-cm margin as Sister Mary Joseph’s nodule in the gastrointestinal surgery department. HCC was controlled with local therapy alone. Three years later, he was referred to the dermatology department for an enlarged lymph node in the left inguinal region. After computed tomography (CT) found no other anomalies, a lymph node dissection was performed (18th July 2023). The metastasis of adnexal carcinoma was found in two lymph nodes. The pathological examination revealed tumor cells containing mucin in the intracytoplasmic, pagetoid spreading in the overlying epidermis of the primary tumor, and immunoexpression of cytokeratin 7, GATA3, and GCDFP15, and negative for hepatocyte paraffin 1 (HepPar1), α-1-fetoprotein, estrogen receptor, CD117, CDX2, all of which fit the diagnosis of invasive EMPD on the umbilical site rather than the umbilical metastasis of HCC (Figures 1b-1d).

**Figure 2:**
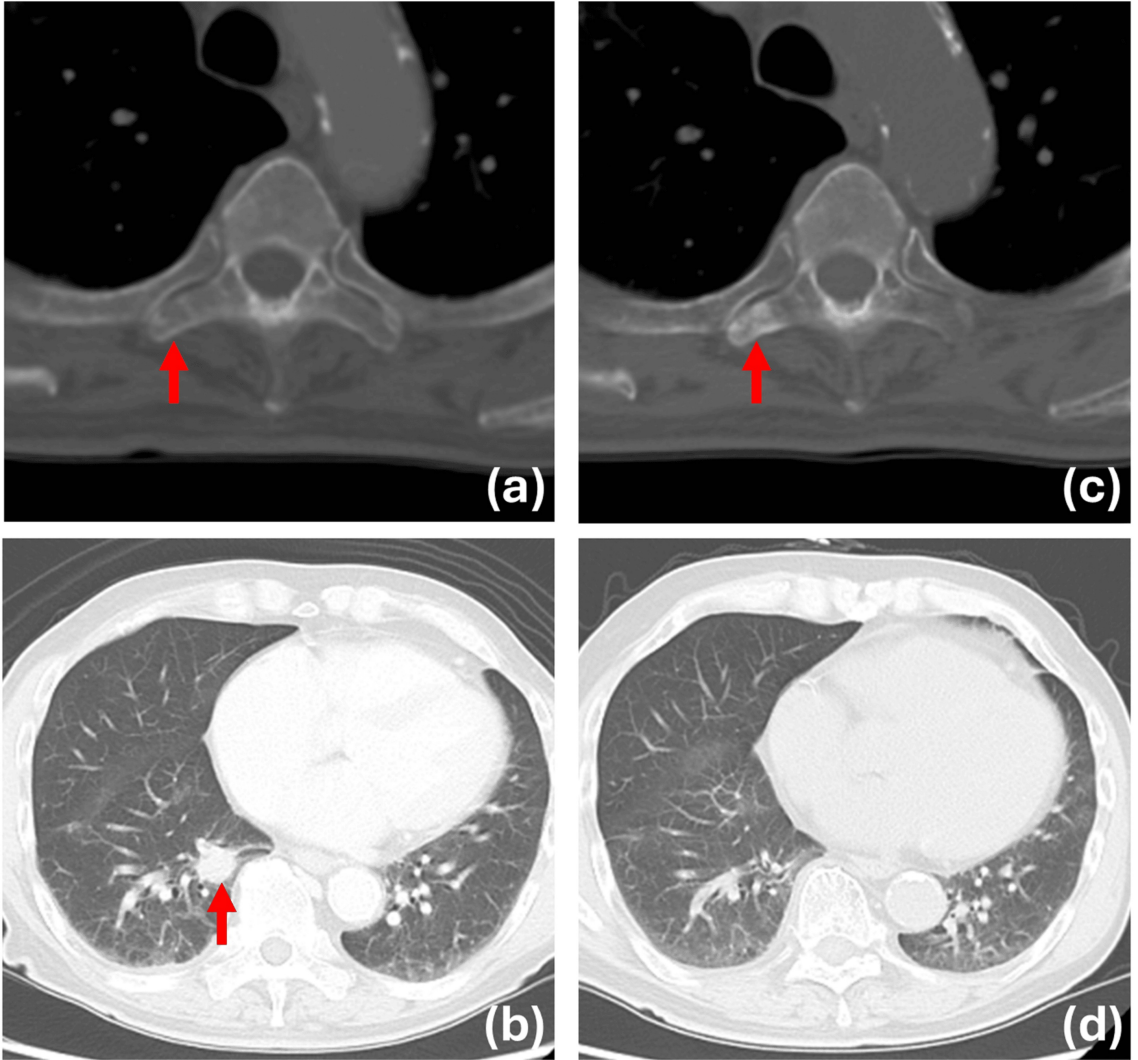
CT findings of the present patient. (a) CT of the thoracic vertebrae before pembrolizumab administration. Osteolytic changes were observed in the right transverse processes of the fifth thoracic vertebra (red arrow), where positron emission tomography-computed tomography (PET-CT) demonstrated accumulation. (b) CT of the lungs before pembrolizumab administration. An 18-mm nodule was found in the right middle lobe (red arrow). (c) CT at month five after the first pembrolizumab administration. The osteolytic changes in the right transverse processes of the fifth thoracic vertebra demonstrated signs of osteosclerosis (red arrow). (d) CT of the lungs at month seven after pembrolizumab administration demonstrated complete resolution of the lung metastases.

No erythema was found on the genitals or in the axilla. Positron emission tomography-CT revealed metastases to the lung and the transverse processes of the fifth thoracic vertebra (17th August 2023). S-1 plus docetaxel therapy was begun. Two months later, CT demonstrated shrinkage of the metastases (partial response) (11th December 2023); however, five months later, the progression of the lesions was evident (Figures 2a, 2b) (30th May 2024). Genetic panel testing (FoundationOne CDx, Foundation Medicine, Inc.) found several genomic alterations (amplification of ERBB2, PARP1, PIK3C2B and RAD51B c.1037-1G>A, CDKN2A p.E88K and p.D108N) and TMB of 12.07 Muts/Mbp, and microsatellite stability. Treatment with pembrolizumab 200 mg Q3W was begun. After the second administration, Grade 3, CTCAE v5.0 acute kidney injury (AKI) developed as a result of ICI-induced tubular necrosis (9th July 2024). The patient was admitted to the hospital for intravenous fluid therapy following a rise in blood urea nitrogen (60.6 mg/dL) and serum creatinine (5.05 mg/dL). Although renal function began to improve within approximately two weeks, it took five months to fully normalize. CT demonstrated progression in multiple lung metastases (9th July 2024), prompting the discontinuation of pembrolizumab therapy. No treatment was given after two doses of pembrolizumab. However, five months after the start of pembrolizumab administration, CT demonstrated shrinkage in the lesions (Figure 2c) (7th October 2024). By month seven, after the start of pembrolizumab administration, all the metastases had disappeared (nearly complete response) (Figure 2d) (20th December 2024). Thereafter, the patient requested that the treatment be discontinued without follow-up.

## Discussion

The present case was marked by findings that were atypical for EMPD. Approximately 92% of lesions occur in areas typically covered by underwear, with involvement of the umbilical region being extremely rare, accounting for less than 1% [4]. In cases where lesions are found in atypical sites such as the umbilicus, it has been reported that multiple lesions, including those in the genital area, may coexist [1]. Clinically, erythema is the most characteristic presentation, and lesions presenting solely as nodules are uncommon [4]. Apocrine carcinoma can present as a nodule in the umbilical region and may be difficult to distinguish from EMPD due to their similar histopathological features [5]. Careful diagnosis is essential, including a thorough examination of the entire tissue architecture to identify pagetoid cells, as well as the use of immunohistochemical staining.

While pembrolizumab is indicated for the treatment of high tissue TMB solid tumors in Japan [6] and 21.5% of EMPD cases have this classification, the drug may not be effective in the latter cases [7]. Moreover, while nivolumab is approved for the treatment of epithelial skin malignancies in Japan, the rate of response of EMPD to the drug was 25%, and there were no cases of CR [7,8]. In addition, a case of advanced EMPD showing a response to pembrolizumab has been reported; however, its effect was temporary [9]. Transient tumor progression after ICI administration has been reported, followed by delayed tumor regression [10]. Therefore, it is important to evaluate the therapeutic effects of ICIs over a longer period. The present report describes a patient with EMPD achieving CR to only two ICI administrations.

Furthermore, the rate of response to ICIs was found to increase when immune-related adverse events (irAEs) occurred [11]. Early incidence of irAEs has been reported to be associated with improved treatment outcomes [12]. Thus, in the present case, the rare irAEs of nephropathy may have been related to the CR following ICI administration. The incidence of AKI due to irAEs related to anti-PD-1 antibody therapy alone is reportedly 2.1% [13]. It has been reported that most cases of ICI-associated AKI occur within the first six months after initiation of ICI, and partial kidney recovery occurred following discontinuation of the ICI [14].

## Conclusions

One limitation of the present report is the fact that recurrences after CR were not evaluated owing to the patient's wish to discontinue therapy. Advanced EMPD is very rare, and there, as of yet, no standard treatment. The efficacy of ICIs against EMPD awaits future investigation enrolling a larger patient cohort.
